# Supplementary material for: Epidemiology of Streptococcus pneumoniae Serotypes in Jordan Amongst Children Younger than the Age of 5: A National Cross-Sectional Study
Source: Vaccines (Basel). 2023 Aug 22;11(9):1396. doi: 10.3390/vaccines11091396 (PMC10536609; doi:10.3390/vaccines11091396)
Supplement: Supplementary file 1 [file vaccines-11-01396-s001.zip › vaccines-2523171-supplementary.pdf]

Supplementary file 1: Details of the 23 culture positive cases

ID.	**Mean age (Months)**	**Serotype**	**Final Diagnosis**	**Presence of chronic illness**	**PCR if done**
R55	4	14	meningitis		positive
R83	53	19A	pneumonia	Yes	positive
R158	4	14	meningitis		positive
R206	4	19F	pneumonia	Yes	positive
R232	8	14	pneumonia		positive
R236	32	19F	pneumonia		positive
KA12	11	6B	pneumonia		positive
kA18	2	18C	meningitis		positive
M1	23	other	pneumonia		positive
M2	33	19F	pneumonia		positive
M3	13	6A	sepsis		positive
M4	6	28	meningitis	Yes	positive
B48	5	1	meningitis		positive
Z64	4	6B	Pneumonia		positive
Z88	35	14	Pneumonia		positive
M8	6	14	Pneumonia		positive
R364	2	19A	meningitis		positive
R528	35	19A	pneumonia		positive
R532	4	18C	pneumonia		positive
R550	3	14	pneumonia		positive
KA27	1	3	pneumonia		positive
B108	12	3	Sepsis		positive
B109	10	14	Sepsis		positive
